# Identification of hereditary cancer in the general population: development and validation of a screening questionnaire for obtaining the family history of cancer

**DOI:** 10.1002/cam4.1210

**Published:** 2017-10-21

**Authors:** Natalia Campacci, Juliana O. de Lima, André L. Carvalho, Rodrigo D. Michelli, Rafael Haikel, Edmundo Mauad, Danilo V. Viana, Matias E. Melendez, Fabiana de L. Vazquez, Cleyton Zanardo, Rui M. Reis, Benedito M. Rossi, Edenir I. Palmero

**Affiliations:** ^1^ Molecular Oncology Research Center Barretos Cancer Hospital Barretos Brazil; ^2^ Center of Molecular Diagnostics Barretos Cancer Hospital Barretos Brazil; ^3^ Oncogenetics Department Barretos Cancer Hospital Barretos Brazil; ^4^ Prevention Department Barretos Cancer Hospital Barretos Brazil; ^5^ Center for Research Support – NAP Barretos Cancer Hospital Barretos Brazil; ^6^ Life and Health Sciences Institute (ICVS) School of Medicine University of Minho Campus Gualtar, Braga, Portugal; ICVS/3B’s‐PT Government Associate Laboratory Braga/Guimarães Portugal; ^7^ Sirio Libanes Hospital São Paulo Brazil; ^8^ Barretos School of Health Sciences Dr. Paulo Prata – FACISB Brazil

**Keywords:** Hereditary cancer, hereditary cancer in low‐income countries, hereditary cancer screening strategies, oncogenetic, pedigree drawing strategies

## Abstract

One of the challenges for Latin American countries is to include in their healthcare systems technologies that can be applied to hereditary cancer detection and management. The aim of the study is to create and validate a questionnaire to identify individuals with possible risk for hereditary cancer predisposition syndromes (HCPS), using different strategies in a Cancer Prevention Service in Brazil. The primary screening questionnaire (PSQ) was developed to identify families at‐risk for HCPS. The PSQ was validated using discrimination measures, and the reproducibility was estimated through kappa coefficient. Patients with at least one affirmative answer had the pedigree drawn using three alternative interview approaches: in‐person, by telephone, or letter. Validation of these approaches was done. Kappa and intraclass correlation coefficients were used to analyze data's reproducibility considering the presence of clinical criteria for HCPS. The PSQ was applied to a convenience sample of 20,000 women of which 3121 (15.6%) answered at least one affirmative question and 1938 had their pedigrees drawn. The PSQ showed sensitivity and specificity scores of 94.4% and 75%, respectively, and a kappa of 0.64. The strategies for pedigree drawing had reproducibility coefficients of 0.976 and 0.850 for the telephone and letter approaches, respectively. Pedigree analysis allowed us to identify 465 individuals (24.0%) fulfilling at least one clinical criterion for HCPS. The PSQ fulfills its function, allowing the identification of HCPS at‐risk families. The use of alternative screening methods may reduce the number of excluded at‐risk individuals/families who live in locations where oncogenetic services are not established.

## Introduction

Approximately, 5–10% of cancer cases have a hereditary pattern, which is caused by germline mutations in oncogenes and tumor suppressor genes 1, 2, 3, 4, 5. The presence of a family history of cancer is one of the most important risk factors for this type of neoplasia 6. Thus, the identification of individuals at‐risk is crucial for the application of proper preventive and therapeutic measures 2, 7 and represents an enormous potential for cancer risk reduction. In addition, the accurate identification of relatives’ wild type for the mutation segregating in an at‐risk family reassures the individual and eliminates the expenses and complications of unnecessary screening and preventive interventions.

One of the challenges for Latin American countries is to introduce hereditary cancer healthcare approaches, with new unexpensive technologies, including screening, genetic counseling, and genetic testing for early detection and diagnosis 8. In Brazil, there are few specialized services for hereditary cancer predisposition syndromes (HCPS) diagnosis and follow‐up 9. These services are primarily available at hospitals attached to medical schools and expensive private institutions, mainly located in major population centers, restricting and prejudicing their access 8, 10. Making matters worse, given the low literacy of a significant proportion of the Brazilian inhabitants 11, part of the general population is unaware of the existence and importance of this type of service 12. Additional reasons for lack of recognition and knowledge regarding hereditary cancer in Brazil include the absence of specialized human resources to meet such demand and the unawareness of the possibility of using family history for cancer prevention 13, despite substantial government programs and the evidence that family history can be used in primary care for disease control 14, 15. As a consequence of all facts exposed above, the attention focused on the screening and identification of individuals and families at‐risk for HCPS remains limited in Brazil and other Latin American countries 16, 17.

Developed countries also face difficulties related to the identification of HCPS families, due to the increasing demand of patients and families at‐risk for hereditary cancer identified and consequent lack of infrastructure and personnel to meet this growing necessity 18, 19, 20, 21. This problem will inevitably occur in developing countries such as Brazil as soon as oncogenetics services become available to the entire population. There is a worldwide need for more cost‐effective strategies to expand the accessibility of Cancer Genetics services without overburdening health services and healthcare professionals 22, 23.

Accordingly, the aim of this study was to create and validate a simple instrument for the identification of families at‐risk for hereditary breast, ovarian, and/or colorectal cancer, as well as to validate different strategies for pedigree drawing of those at‐risk individuals identified.

## Methods

### The primary screening questionnaire

The primary screening questionnaire (PSQ) intents to be an easy and quick way to identify individuals and/or families probably at‐risk for HCPS, mainly focusing on breast, ovarian, and/or colorectal cancer predisposition syndromes.

The PSQ contains information regarding the age of cancer diagnosis and number of tumors in the family, which are the basis of most clinical criteria, such as ASCO 24, 25, NCCN 26, and Amsterdam 27. In addition, because breast and colorectal cancers are present in a higher frequency in our population, a specific question addressing these two tumor types were also included.

The questionnaire was structured to have a YES/NO answer for the following questions: Q1—Personal history of cancer before the age of 50 years; Q2A—First‐ or second‐degree relatives with breast cancer (BC) before the age of 50; Q2B—First‐ or second‐degree relatives with ovarian cancer (OC); Q2C—First‐ or second‐degree relatives with colorectal cancer (CRC) before the age of 50; and Q3—Three or more first‐ or second‐degree relatives with cancer.

### Population of the study

The PSQ was applied in a convenience sample of 20,000 women from Barretos Cancer Hospital (BCH) during a period of 12 months. Women submitted to a routine mammogram or cervical cancer screening were invited to participate in the study regardless of her personal or family cancer history.

The Cancer Prevention Unit at BCH (Barretos, Brazil) performs population‐based preventive cancer screening and attends more than 100,000 individuals per year. The Institution also has 12 Mobile Units, which perform prevention screening regarding breast, cervix, prostate, and skin cancer all over the Brazilian territory, reaching remote areas 28, 29, 30. For the purpose of the present study, women performing mammogram or cervical cancer screening at the Cancer Prevention Unit at BCH or, also, in the Mobile Units that cover São Paulo state, were invited to participate.

### Secondary screening

All women who answered affirmatively to at least one of the PSQ questions were invited to participate in a second phase of the study. This secondary screening involved a further analysis of the family cancer history with pedigree drawing (including at least three generations), encompassing maternal and paternal family history. A properly trained healthcare professional (nurse with experience in Cancer Genetics) conducted the second phase, using three different approaches, as described below.

#### In‐person approach

After informed consent fulfilling, the appointment consisted of an initial conversation to provide a general explanation about the importance of cancer prevention and early detection. At this moment, educational materials regarding cancer prevention and genetic risk factors associated with cancer development were provided to the participant 12. Next, the pedigree was drawn. The language used throughout the process was simple and easily comprehensible for all patients independent of the education/literacy level.

#### Telephone approach

In this approach, the pedigree was created from information obtained by telephone and by the same interviewer who conducted the in‐person interviews. Telephone conversations lasted 30–40 min. Once again, the language was simple and easily comprehensible for participants with different education levels. After hearing the informed consent (by telephone), the participant could decline to participate in the study and end the telephone call if desired.

This approach was performed for those women reporting unavailability to visit the health service, due either to work or domestic activities.

#### Letter approach

In this approach, an envelope with the following items was mailed to the participants: (1) a letter that explained the research project and what procedures were involved in the second phase of the study; (2) two copies of the informed consent; (3) a questionnaire with several questions regarding the family history of cancer in the first‐, second‐, and third‐degree relatives, including the type of cancer, age at diagnosis, current age or age at death, the presence of multiple or bilateral tumors, and the relationship of the cancer patient to the person who was filling out the questionnaire (the questionnaire was adapted from a model of the Brazilian Network of Familial Cancer); and (4) a stamped envelope, so the participant could return the questionnaire and the informed consent, free of charge, by mail.

The letter approach was used for those women who could not be reached by telephone (had no telephone number or did not answer the telephone).

### Primary screening questionnaire validation

The sensitivity, specificity, positive, and negative predictive values of the PSQ were evaluated using the kappa coefficient.

To calculate the sample size needed for this validation, the presence of a positive family history of cancer was considered. In the case of a positive PSQ, where the expected ratio is 0.80, with a significance of 0.05, absolute error of 0.09 in the confidence interval, and the population size of 1938 (number of women who answered positively in our study and had the pedigree drawn), the validation would require about 69 cases. In the same way, it was possible to identify the proportion of no family history of cancer for those who answered “NO” in the PSQ. Thus, for the negative group of the PSQ (population size of 16,879 women), taking into account the expected ratio of 0.80, a significance of 0.05, absolute error 0.075, and population size in the confidence interval, 107 cases would be necessary.

Based on these estimates, a sample of 69 women with at least one affirmative answer to the PSQ and 107 women with negative responses to all PSQ questions were randomized. Pedigrees were drawn for all 176 cases. In addition, the PSQ was reapplied 6 months after the first application. At each stage, the questionnaires were applied independently, meaning that the interviewers did not know the answers of previous applications.

### Validation of pedigree drawing

The sample size for validation of the approaches (in person, by telephone, and letter) was estimated based on the reproducibility of clinical criteria using the table published by Sim & Wright 31. An expected kappa of 0.8, alpha of 0.05, and a power of 0.9 with a kappa coefficient under the null hypothesis of 0.4 were considered. Additionally, considering that the proportion of positive cases of HCPS at the study population was of 0.3, around 60 cases were needed. The in‐person approach was considered as the *gold standard* strategy. To validate the telephone approach, 60 cases in which the pedigree was created from information obtained “in‐person” were randomly selected. Then, telephone calls were made to these subjects. These confirmation calls were not performed by the same person who performed the in‐person interview, and the callers had no access at the time of the interview or earlier to the previously created pedigrees.

The validation was also performed for the letter approach. A total of 60 cases whose pedigree was initially created from information obtained by letter were analyzed and systematically compared with the telephone approach once this approach was validated.

To verify the reproducibility of the approaches, kappa (for categorical variables) and intraclass correlation coefficients (for numerical variables) were calculated, by comparing the following variables: fulfillment of HCPS clinical criteria (yes/no, and if yes, which criteria), number of generations, number of generations affected by cancer, total number of tumors, number of first‐ and second‐degree relatives with cancer, and those with cancer before the age of 50.

Regarding fulfilling HCPS criteria, the clinical criteria for the main hereditary breast and/or colorectal cancer predisposition syndromes, such as hereditary breast ovarian cancer (HBOC) 26, Li–Fraumeni syndrome (LFS) 32, hereditary breast and colorectal cancer (HBCC) 24, 25, familial adenomatous polyposis—FAP 3 (considering the presence of personal or family history of polyps or previous polypectomy), and Lynch Syndrome 27, were considered.

### Referral for specialized Cancer Genetics service

All families that fulfilled at least one clinical criterion for any HCPS are being referred to the Oncogenetics Department of BCH, and, after genetic counseling and family history confirmation, they are referred to the Center of Molecular Diagnosis of the same Institution for genetic testing, if warranted 9.

### Ethical aspects

All the participants completed the informed consent. The study was approved by the Research Ethics Committee of the BCH, Sao Paulo state, Brazil, under protocol number 413/2010.

## Results

### The primary screening questionnaire

From the total sample of 20,000 women enrolled, 17,092 were from the Mobile Units and 2909 from the Hospital Unit. They were from 381 cities distributed along 20 different Brazilian states. The average age was 51 years (SD = 9.45), ranging from 18 to 79 years of age. As it would be expected, since the participants were women performing regular mammogram and/or cervical cancer screening, the great majority of the participants (91.0%) were older than 40 years.

From all of them, 3121 (15.6%) answered affirmatively to at least one PSQ question. The most frequent positive answer was regarding the presence of first‐ or second‐degree relatives with BC before the age of 50 (*n* = 1438, 46.0% of the respondents). Detailed information, question by question, can be found in Table 1.

**Table 1:** Number of positive answers for questions in PSQ

	N	%^a^
Q1—Personal history of cancer before the age of 50 years old;	554	17.7
Q2A—First‐ or second‐degree relatives with BC before the age of 50;	1438	46.0
Q2B—First‐ or second‐degree relatives with OC;	662	21.0
Q2C—First‐ or second‐degree relatives with CRC before the age of 50;	456	14.6
Q3—Three or more first/second‐degree relatives with cancer.	800	25.6

a One participant can answer affirmatively to more than one question in PSQ.

### Secondary screening

Among the 20,000 women who completed the PSQ, 3121 (15.6%) had at least one positive answer and were invited to the second phase of the study. During the second part of the screening, there was an attempted to approach each of the 3121 women in order to draw their pedigree. Of all the participants (3121), 17 were unable to participate in the study (6 died and 11 had psychological problems and their relatives did not want to respond), 79 directly verbalized that they did not want to participate, and a total of 1087 were unsuccessfully attempted to be contacted by telephone or letter. In order to eliminate the possibility of bias due to the number of participants that were unable to be contacted, chi‐square test was realized to compare the profile of answers in PSQ in the group that was possible to reach and had the pedigree analysis with the group that was unable to be contacted. The result of this analysis shows that the profile of affirmative responses is similar, reducing the possibility that we have recruited only those of higher risk or those of lower risk.

**Figure 1:**
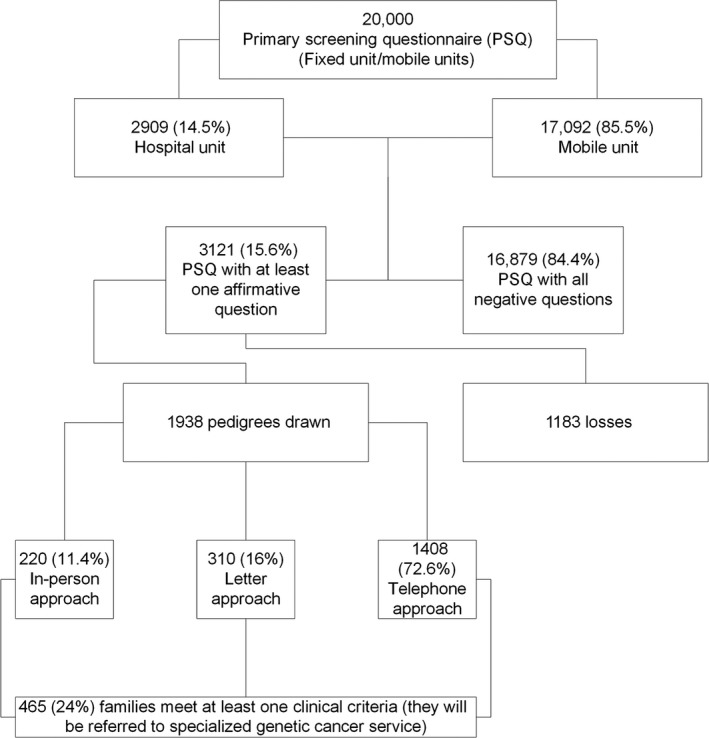
Flowchart.

In total, 1938 women (62.1% of the total sample) were reached, and the pedigree for each of them was drawn. Using the “in‐person” approach, we were able to reach and draw 220 (11.3%) pedigrees, while 310 (16.0%) and 1408 (72.6%) women were reached by letter and telephone, respectively. Figure 1 depicts the study phases with the number of participants enrolled in each phase.

We conducted a detailed analysis of all 1938 obtained pedigrees. The number of cancer‐affected probands was 200 (10.3%) excluding skin nonmelanoma cases, and the most frequent type of cancer was breast (66 cases, 33.0%), cervix/uterus (47 cases, 23.5%), and thyroid cancer (20 cases, 10.0%). The families evaluated showed a large number of relatives, ranging from 2 to 30 relatives of first degree and 4–27 of second degree. The mean number of tumors was two per family (SD = 1.67; 95% CI: 0–13). Considering the tumor spectrum present in those families, some interesting findings are depicted in Table 2. Ninety‐nine families with BC cases before the age of 35 were identified, and among them, six families had more than one of those cases in their families.

**Table 2:** Frequency of the type of the tumor in the analyzed families

Type of cancer	General frequency	General %	Number of cases in the same family	Relative frequency	Relative %
Breast cancer before 35 years old	99/1938	5.1	1	93/99	93.9
			2	6/99	6.1
Bilateral breast cancer	17/1938	0.9	1	17/17	100
			2	–	–
Male breast cancer	1/1938	0.05	1	1/1	100
			2	–	–
Colorectal cancer before 50 years old	195/1938	10.0	1	171/195	87.7
			2	24/195	12.3
Pancreatic cancer	44/1938	2.3	1	44/44	100
			2	–	–
Ovarian cancer	39/1938	2.0	1	38/39	97.4
			2	1/39	2.6

Regarding clinical criterion for HCPS, the following guidelines were considered: ASCO 24, NCCN 26, Amsterdam 33, Bethesda 27, and Li–Fraumeni (classic proposed by Li and Fraumeni and, for Li Fraumeni like, those proposed by Birch and by Chompret) 32, 34. In addition, the criteria proposed by the Brazilian Institute of Cancer (for polyposis) were also considered 3.

Among the 1938 families carefully analyzed, 465 (24.0%) had at least one clinical criterion; 402 (20.7%) cases had only one criterion present in the family; 57 (2.9%) fulfilled two different criteria in the family; and 6 (0.3%) had three criteria identified. The results obtained are consistent with the literature, where the less stringent criteria were the more frequently observed: 273 families fulfilling the NCCN criteria for HBOC syndrome 26 and 195 families with the Bethesda criteria for Lynch syndrome 27. Table 3 sets out the main clinical criteria observed.

**Table 3:** Number of families per clinical criteria^a^

Clinical criteria	N^b^	%
HBOC (ASCO criteria)	37	2.0
HBOC (NCCN criteria)	273	14.1
Lynch syndrome (Amsterdam criteria)	13	0.7
Lynch syndrome (Bethesda criteria)	195	10.0
Li–Fraumeni like (Birch criteria)	11	0.6
Li–Fraumeni like (Chompret criteria)	8	0.4
Cowden syndrome (CCOD criteria)	19	1.0
Familial adenomatous polyposis (INCA criteria)	6	0.3

HBOC, hereditary breast and ovarian cancer; ASCO, American Society of Clinical Oncology; NCCN, National Comprehensive Cancer Network; CCOD, cowden consortium operational diagnoses; INCA, Brazilian National Cancer Institute—National Network of Familial Cancer, Brazil.

a The percentage value was calculated considering the number of families per clinical criteria from a total of 1938 that had the pedigree drawn.

b Some families could had more than one clinical criterion.

### Validations

#### Primary screening questionnaire

**Figure 2:**
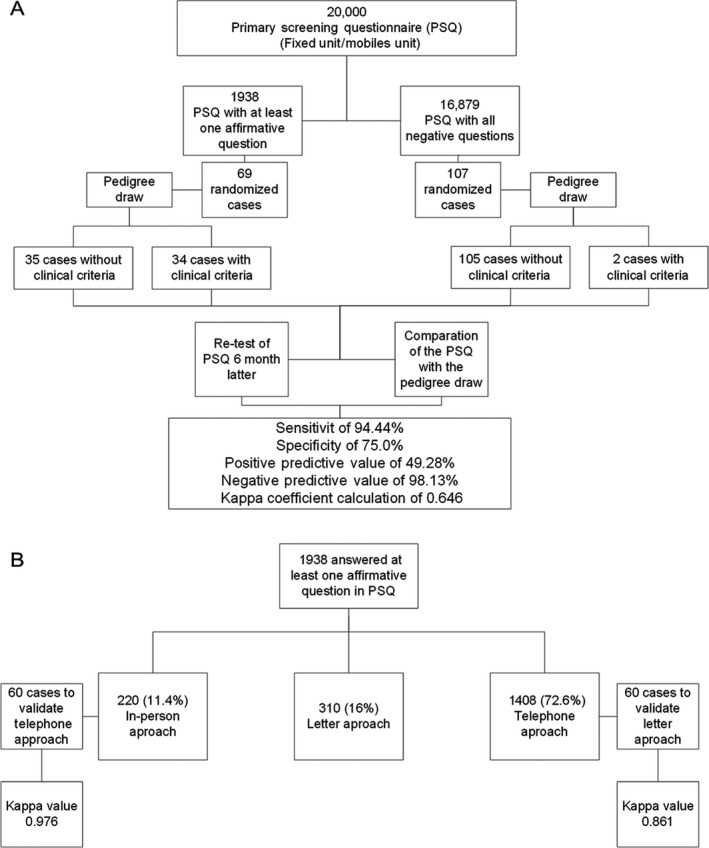
Validation approaches. (A) PSQ validation. (B) Telephone and letter validation.

The PSQ validity was measured by the sensitivity, specificity, positive and negative predictive values, as well as through the calculation of kappa coefficient for the comparison with pedigree data (Fig. 2A). The sensitivity and specificity rates of the PSQ to identify families with clinical criteria for an HCPS were 94.4% (95% CI [0.813–0.993]) and 75% (95% CI [0.669–0.818]), respectively. The positive predictive value of the instrument was 49.3% (95% CI [0.419–0.566]), and the negative predictive value was 98.1% (95% CI [0.931–0.995]), showing that the PSQ satisfies the real necessity for screening families at potential risk (Table S1 shows the rationale to calculate sensitivity, specificity, and positive predictive value and negative predictive value).

The reproducibility of the PSQ considering an interval of 6 months between application and reapplication was also evaluated. The kappa value obtained was 0.646 (SD = 0.05, *P* < 0.001), presenting good concordance between both time points, pointing to the accuracy of self‐reported personal and family history.

#### Approaches for obtaining cancer family history

Considering the difficulties we faced to have all participants coming to the Hospital Unit for the secondary screening phase, alternative strategies were employed to obtaining their pedigree. All strategies were validated considering, as detailed in the methodology, the “in person” strategy as the gold standard. Figure 2B demonstrates a flowchart that simplifies the process of validation.

##### Telephone approach validation

Considering the reproducibility among the variables in the two approaches analyzed: in‐person and telephone (Table 4), the kappa coefficient was calculated and, considering the presence of clinical criteria for any HCPS, a highly reproducible kappa value of 0.976 (error value of 0.023) was obtained. This outcome demonstrates that the completion of this questionnaire by telephone is feasible and reliable.

**Table 4:** Kappa values for reproducibility of the telephone approach compared with the in‐person approach and letter approach compared with the telephone approach

	Coefficient of reproducibility and concordance	
Variable	Telephone approach	Letter approach
Clinical criteria	0.976^b^ (95% CI [0.930–1])	0.850^b^ (95% CI [0.709–0.991])
Number of generations	0.259^b^ (95% CI [−0.235–0.556])	0.641^b^ (95% CI [0.217–1])
Number of affected generations	0.699^b^ (95% CI [0.497–0.820])	0.694^b^ (95% CI [0.565–0.824])
Number of first‐degree relatives	0.962^b^ (95% CI [0.936–0.977])	0.920^b^ (95% CI [0.870–0.970])
Number of first‐degree relatives with cancer before age 50	0.824^b^ (95% CI [0.706–0.895])	0.827^b^ (95% CI [0.702–0.953])
Number of second‐degree relatives	0.824^b^ (95 CI [0.706–0.895])	0.768^b^ (95% CI [0.639–0.897])
Number of second‐degree relatives with cancer before age 50	0.828^b^ (95% CI [0.707–0.899])	0.595^b^ (95% CI [0.312–0.879])
Total number of tumors	0.806^a^ (95% CI [0.673–0.884])	0.861^a^ (95% CI [0.776–0.915])

CI, confidence interval.

a Intraclass correlation coefficient: calculated because it was a numerical variable.

b Kappa.

##### Letter approach validation

The validation of the letter approach was performed by telephone, depending on the availability of the 60 cases randomly selected for the validation. This analysis occurred after the telephone approach validation. This procedure was performed by three different professionals who had no access to the previously created pedigrees.

Table 4 shows the reproducibility values obtained for the different variables considered. Considering the presence of clinical criteria, a kappa value of 0.850 was obtained, which is considered a highly reproducible value 31.

Concordance of clinical criteria fulfillment, calculated from pedigrees constructed with the in person, telephone, or letter, showed that all three approaches were reliable to identify individuals and/or families at‐risk for HPCS.

## Discussion and Conclusion

The PSQ had high sensitivity (94.4%) and specificity (75%), showing that it is an effective tool for identifying individuals and/or families at‐risk for hereditary cancer in a population‐based sample. In fact, taking into account that the PSQ was not strict and that HCPS are relatively rare, the NPV (98.1%) revealed that few families at‐risk for hereditary cancer were missed. The PSQ reproducibility was good (kappa coefficient of 0.646), which is a characteristic of a precise and consistent screening tool. It is possible that the kappa coefficient was not higher because family history is dynamic and can change along time.

Ashton‐Prolla and colleagues previously developed and applied a questionnaire with seven questions related to breast, ovarian, and colorectal cancer family history in the South of Brazil 35. The authors analyzed a total of 9218 participants, of which 885 (9.6%) provided a positive response regarding a personal and/or family history of cancer. From those, 211 women (23.8%) exhibited a suggestive family history of breast and colorectal predisposition syndromes. When comparing both methods, we detected two main differences: (1) The seven‐question questionnaire developed by Ashton‐Prolla is more HBOC/HBCC‐directed than the PSQ described here, and (2) the questionnaires were administered in different Brazilian states with distinct populations and characteristics. However, the percentage of at‐risk families identified was similar.

Regarding secondary screening data (pedigree analysis), the “presence of clinical criteria for HCPS” was chosen as the “comparison variable” for the analysis of information reproducibility, because most of the evaluated variables (e.g., age at cancer diagnosis, number of generations and cancer cases in the family) belong to the clinical criteria for the majority of HCPS. Thus, considering the presence of clinical criteria (in the family) as reference, high reproducibility for the telephone (0.976) and letter approaches (0.850) was obtained, which demonstrated that the most important aspects of the family history of cancer could be obtained through both strategies. Therefore, the reliability of the obtained information 15 and the validity of these approaches for identifying families at‐risk for hereditary cancer were demonstrated. Results obtained in this study may generate several healthcare benefits: (1) increase access to highly specialized services with Hereditary Cancer Genetics department, (2) reach a specific population that is not able to go to the health services, (3) reduction of stress, costs, and time (required for travel to the first consultation), (4) promote efficient communication between health services and their users, and (5) train health system managers/governors aware of the importance of creating and maintaining this type of specialized service in Genetics and Cancer.

Several studies report the use of alternative methods for patient access 19, 36, 37. Pieper et al. 19. evaluated a questionnaire to screen individuals at‐risk for hereditary CRC in a sample of private service users in Germany. A questionnaire on the presence of a family history of CRC was mailed to 12,139 individuals. Of these, 2355 individuals completed and returned the questionnaire, and it was verified that 373 (16%) of the participants had some risk for hereditary CRC. A study developed by Joseph and colleagues 36 had a screening questionnaire that was applied by telephone, and, according to the answers, it was possible to score it and verify if the person was eligible for genetic counseling. Other authors 37, 4 years later, used the same methodology to screen by telephone individuals eligible for genetic counseling. The studies cited above identified high‐risk families that could participate in different strategies of cancer risk assessment.

In addition to screening strategies, new alternatives have been used for the genetic counseling process. In a study by Platten et al., genetic counseling was performed by telephone to identify individuals at‐risk and facilitate their assistance 23. It was observed that the patient's service satisfaction and feelings regarding the care provided by this type of approach had the same outcomes as observed in a conventional method. Another study published by Kinney and collaborators in 2016 compared genetic counseling by telephone with the in‐person strategy for individuals that live in geographically diverse areas and concluded that telephone counseling can be effectively used to increase access without long‐term adverse psychosocial consequences 38.

An important issue to address is the importance of the educational strategies, once this is a general population that may have never listen about “Oncogenetics” or “Hereditary Cancer” 12. Educational tools can be developed to help the process like, for example, a brochure that can be delivered during the prevention exams and can help on the understanding of the associated concepts and also contribute to reduce the stress concerning the first consultation on Genetics department.

We could, with the present study, identify that, among those who answered positively in the PSQ, 24% (465 women) had at least one clinical criterion for HCPS. Even though the prevalence of HCPS cannot be inferred from this study, the prevalence in a selected sample can still add important information for cancer programs planning. All at‐risk identified cases are being referred to the Oncogenetics department for cancer genetic counseling and, for those families with criteria for genetic testing, the same is performed without any charge to the patient. It is important to emphasize that there is a very low probability that this group of women would have had the opportunity to have a consultation with a geneticist outside the context of the present study, revealing the necessity of screening strategies in countries of lower socioeconomic level.

Regarding difficulties faced during the study, we can point out the problem to access the entire population. We had a loss of 38% in the sample group that answered at least one affirmative question in PSQ (3121). This can be justified by the fact that most of the participants were from remote areas (inner region of the country). In addition, the great majority is economically active, which makes harder the possibility of reaching health services.

This study has some limitations as the fact that, with few exceptions, the tumor diagnosis was not confirmed by death certificates or pathology reports. Besides, sometimes, the age at cancer diagnoses was not properly considered at the time of PSQ application. When comparing the PSQ answers with the pedigrees, we could see that if the participant had a previous diagnosis of cancer, she got a “YES” in the first PSQ question, regardless of age at diagnosis. The same is true for answers is questions Q2A to Q2C. Another limitation that should be addressed is the fact that some cases of OC may have been lost if they were diagnosed over the age of 50 in the study participants.

In conclusion, it is worth noting the importance of using alternative forms of patient access and identification of families at‐risk for hereditary cancer. Considering the extensive Brazilian geographic area, the scarcity of oncogenetics services, and the concentration of these services in a few cities, Brazil (and other countries with the same reality), there is a need for different strategies to reduce disparities in healthcare access 39 and new strategies for cancer prevention. The current study demonstrates, in a simple way, the high quality of the information obtained through unconventional approaches for pedigree drawing. These approaches can be used to assemble a detailed and reliable family history and identify (even in geographically remote areas) families at‐risk for hereditary cancer. The information obtained will be useful to refer such families to appropriate preventive programs according to their risk, thus reducing the risk of cancer and increasing the possibility of a real personalized medicine.

## Practice Implications

The process of screening individuals and families who may be at‐risk could be more economical and faster, particularly where the healthcare delivery system remains underdeveloped and where problems occur in the organization and delivery of preventive practices.

The achievement of this simple questionnaire (PSQ) during screening tests can be highly effective since the patient is already present in the preventive care environment, not restricting only to populations that have already been diagnosed with cancer or those already considered of high risk.

## Conflict of Interest

We hereby declare that we have no conflict of interest.

## Supporting information


**Table S1.** Response to PSQ questions and fulfillment of clinical criteria for HCPS*.
