# Supplementary material for: Identification of hereditary cancer in the general population: development and validation of a screening questionnaire for obtaining the family history of cancer
Source: Cancer Med. 2017 Oct 21;6(12):3014–24. doi: 10.1002/cam4.1210 (PMC5727305; doi:10.1002/cam4.1210)
Supplement: Supplementary file 1 — Table S1. Response to PSQ questions and fulfillment of clinical criteria for HCPS*. [file CAM4-6-3014-s001.docx]

Supplementary table1: Response to PSQ questions and fulfillment of clinical criteria for HCPS*.

|  | | **Clinical Criteria** | |  |
| --- | --- | --- | --- | --- |
|  |  | **No** | **Yes** | **Total** |
| **Randomized groups** |  |  |  |  |
| Participants who answered NO to the PSQ |  | 105 | 2 | 107 |
| Participants who answered at least one YES to the PSQ |  | 35 | 34 | 69 |
| **Total** |  | 140 | 36 | 176 |

*These value were used for sensitivity, specificity, NPV and PPV calculations: Sensitivity: 94% (95% CI [81.3% - 99.3%]; Specificity: 75% (95% CI [66.9% - 81.93%]); NPV: 98% (95% CI [93.1% - 99.5%]); PPV: 49% (95% CI [41.9% – 56.5%]).
